# Augmented future: tracing the trajectory of location-based augmented reality gaming for the next ten years

**DOI:** 10.1515/icom-2024-0018

**Published:** 2024-05-14

**Authors:** Samuli Laato, Heinrich Söbke, Manuel F. Baer

**Affiliations:** Gamification Group, 7840 Tampere University, Tampere, Finland; Hochschule Weserbergland, Hameln, Germany; 26597 Bauhaus-Universität Weimar, Bauhaus-Institute for Infrastructure Solutions (b.is), Weimar, Germany; Centre for eResearch, 1415 University of Auckland, Auckland, New Zealand

**Keywords:** locative media, location-based games, augmented reality, trends, review

## Abstract

Location-based games are a highly technology-dependent game genre that has witnessed an exponential increase in popularity with the democratisation of smartphones as well as ubiquitous mobile data and access to satellite navigation. Moving forward into the future, location-based games can be expected to evolve as the technologies underlying the genre improve. In this conceptual work, we review the current state of the art in location-based games, and identify key trajectories and trends. We discovered 12 trends, based on which we jump ten years into the future and evaluate how current technology trends may end up influencing location-based gaming. For example, we propose that in the year 2035 through improvements in map data services and sensor data coverage, we will see locative games that are increasingly connected to elements in the physical world. We also expect to see gameplay that moves away from solely taking place on a smartphone screen to the adoption of multiple forms of interactions with location-based game worlds, especially as head-mounted displays and other wearables become more commonplace.

## 1 Introduction

Location-based games (LBGs) are a genre of mobile gaming where the player’s physical location is a central part of the playing experience.^1^ Examples include Niantic’s Ingress^2^ and Pokémon GO,^3^ Ludia’s Jurassic World Alive^4^ and Meyran Games’ Plant the World.^5^ All these LBGs offer players a map interface based on the real world, that is augmented with fictional and playful content. Over the years, LBGs have also been discussed under the umbrella of various other terms including location-based augmented reality (AR) games, geo games, hybrid reality games, locative games and pervasive games, all carrying different nuances and connections to different research traditions.^6^ In this work, we stick with the most popularly used term LBG.

According to Statista,^7^ the most popular LBGs such as Pokémon GO and Dragonquest Walk make triple digit millions of USD profit a year in in-app purchases, and they have monthly active users in the tens of millions. The popularity of the genre does not guarantee success for individual games, as LBGs such as NBA All World^8^ and The Witcher Monster Slayer^9^ have been promptly shut down and discontinued shortly after their release. Due to these setbacks, there is a need to reappraise the LBG genre in light of recent technological and design innovations. Some of these innovations are design-related, such as the Routes feature in Pokémon GO, the paintball feature in Monster Hunter Now, or the building mechanics in Orna. Others relate to more technical aspects, such as map data, sensors and gaming hardware. Taken together, these trends can be seen as paving the way for new immersive AR experiences.^1^ ^,^ ^10^

Therefore, the aim of this work is to review the academic literature on factors related to LBGs, and extract extant paramount trends for the future of LBGs. We focus our review and analysis on three key areas of research and developments: (1) geographic information systems (GIS) and map services; (2) technical infrastructure and hardware; and (3) design issues. After the discovery of trends pertaining to these three dimensions, we then analyse how they are likely to impact the future of location-based gaming. In other words, this work presents an evidence-based speculation of the future of LBGs based on trends that are visible in the extant scholarship on the genre. To guide this research, we propose the following research questions (RQ).

– **RQ1: What trends exist in locative gaming today based on the extant academic scholarship?**
– **RQ2: In the authors’ interpretive view, what does the future of LBGs look like in ten years based on the observed trends?**

The rest of this study is structured as follows. First, we review and present the current state of the art of location-based gaming research. We extract key trends, and then offer our view of the future of locative gaming and present concrete speculative design examples. Finally, we discuss this study’s contributions, the limitations of this work and propose future research avenues.

## 2 Trends relevant for location-based gaming – a review of the literature

LBGs can be regarded as a highly technology-dependent genre. Contrary to PC and console gaming, successful LBGs require a mobile internet connection, a backend of map data services and they make use of sensor data such as satellite navigation,^11^ gyroscopes, camera and LiDAR for e.g., positioning augmented reality (AR) content.^12^ We list the key similarities and differences in the technical requirements between console/desktop gaming, mobile gaming and locative gaming in Table 1. While the differences are not always clear, e.g., some mobile games do not require a stable internet connection and some offline games can still make use of sensor data (think of Ring Fit Adventure as an example^13^), it is still evident that LBGs rely on a wide range of technologies, involving both hardware and software.

**Table 1:** The technological dimensions involved in LBGs, mobile games and console/PC multiplayer online and offline games. This table provides an idea of why locative gaming is such a technology-dependent genre.

Technical requirement	LBGs	Other mobile games	Console/PC (online)	Console/PC (offline)
Stable internet connection	Important	In most cases	In most cases	N/A
Mobile internet connection	Important	Depends	N/A	N/A
Client-side computing	Important	Important	Important	Important
Server side computing	Important	Depends	In most cases	N/A
Map data services	Important	N/A	N/A	N/A
Sensor data	Important	Depends	N/A	In a few cases

Moving beyond the technical requirements, there are also various other challenges intrinsic to LBGs such as design and privacy challenges^12^ as well as challenges pertaining to elements in the real world.^1^ There have been studies focusing on these aspects specifically, such as studies on how LBGs incentivise movement,^14^ how to design for social interaction in LBGs^15^ ^–^ ^17^ and how to protect LBG players’ privacy.^18^ In the following we begin by reviewing the technical trends and trajectories, which we divide into two key categories: (1) trends in GIS and map services; and (2) trends in technical infrastructure and hardware. We then finally discuss the trends in (3) design issues.

### 2.1 Trends in geographical information systems and map services

Geographic information systems (GIS) and map services such Google Maps (launched in 2005) and OpenStreetMaps (launched in 2004) have already provided navigation services and geographical information for roughly two decades. These map services make use of satellite navigation (such as the Global Positioning System (GPS) or Globaluaya Navigatsionnaya Sputnikovaya Sistema (GLONASS)^11^) to position the user in a real-world map. These systems are widely used by laypeople for activities such as travel and tourism. All these services also increasingly contain information about the places and spaces that they describe. For example, Google Maps contains descriptive information (e.g. coordinates, contact details and opening hours) as well as perceived information (e.g. customer reviews) concerning real-world points of interest such as cafés, restaurants, tourist attractions, places of worship and libraries.^19^ ^,^ ^20^ In other words, these map services now make use of user-generated content to bring meaning, information and understanding of places and spaces.^21^ Therefore, we summarise the following trend.

**Figure 1:**
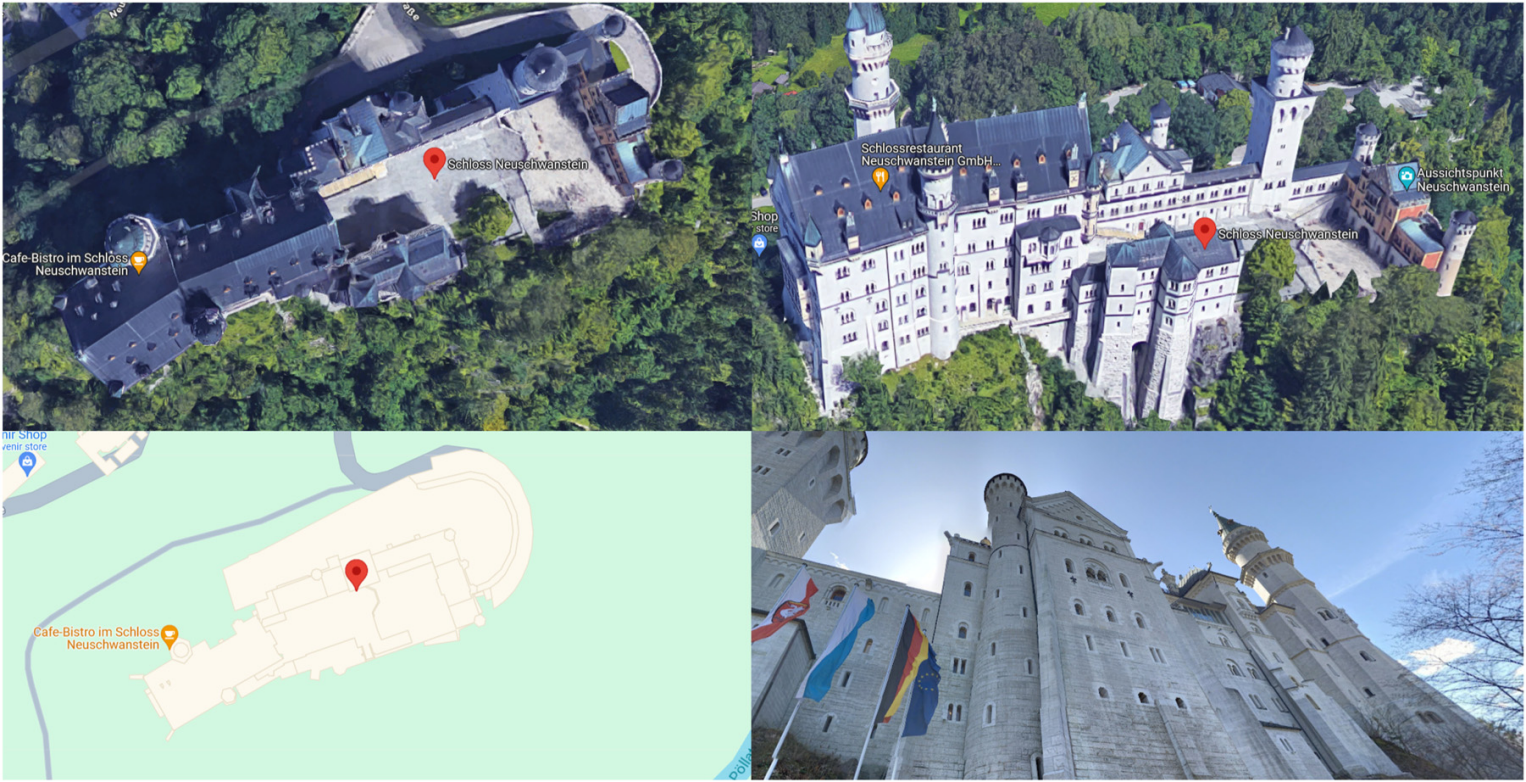
Four screenshots taken by the authors from Google Maps. The photos showcase the Neuschwanstein Castle from a top-down satellite view (top left), an angled composite view (top right), a traditional abstract map view (bottom left) and Google street view (bottom right).

– **(GIS) Trend #1:** *Spatial data providers and map data services are increasingly augmented with user-generated content including objective descriptive data as well as subjective individual perceptions of environments and services.*
  Further, spatial data acquisition and visualisation have seen a steady increase in fidelity. In Figure 1, we display four views of the same location in Google Maps, each a separate layer in the system affording different interactions and allowing the user to tailor their experiences to their needs. Over the past two decades, (online) mapping services have transitioned from using raster image tiles, to vector tiles, with the latest development introducing high-fidelity photorealistic 3D map tiles1For more information, see https://developers.google.com/maps/documentation/tile/3d-tiles (accessed: 17.02.2024). (top right in Figure 1). This trend towards higher fidelity 3D spatial information has been accelerated by the advances in image-stitching techniques^22^ in combination with the emergence of affordable unmanned aerial vehicles (UAVs) featuring high-quality cameras, allowing for large geographic extents to be mapped in three dimensions at relatively low costs.^23^ However, we should also note that high-fidelity photorealistic spatial data is not available globally and areas of low spatial data quality still exist in large quantities,^24^ ^–^ ^26^ which is in line with findings showing underrepresented digital content in certain geographic areas in LBGs.^27^ ^–^ ^30^ Nonetheless, we can see a clear trend towards higher fidelity map data and we accordingly summarise the following trend.
– **(GIS) Trend #2:** *Spatial data providers and map data services are offering spatial information with increasing fidelity resulting in real-world places being depicted on maps with increasing precision, levels of detail and real-world information.*
  Together with user-generated content such as customer reviews being embedded in map services,^21^ and the map data becoming available in higher fidelity, information about a specific geospatial entity is being recorded with increasing levels of detail. Instead of defining a spatially situated entity as a building, a park or a road, information with varying levels of granularity is being recorded and attached to these entities. This allows us to identify, for example, the type of building (e.g. castle, school, hospital), the recreational facilities available in a park (e.g. playground, outdoor gym, running track) and specific mobility-related information (e.g. material, width, curb height) of roads, effectively moving from broader categories of spatial entities to highly-detailed machine readable information. This trend has been supported by emergent modern machine vision techniques as well as user-generated content leading to the identification of map content with higher accuracy.^31^ Increasingly detailed and available meta-data on real-world entities has far-reaching implications in various domains such as urban planning,^32^ ^,^ ^33^ ecosystem services research^34^ ^,^ ^35^ as well as in the generation and accurate positioning of fictional game content in location-based games.^1^ Thus, we propose a third trend in the area of GIS.
– **(GIS) Trend #3:** *Information about real-world spatially situated entities is being recorded and made available with increasing levels of granularity in machine-readable formats, in particular in terms of the categories an entity belongs to.*
  A unique requirement for globally available and enjoyable LBGs is the global availability of game-relevant content, and spatial information that serves as the seed for generating the digital game content. Such spatial data commonly comes from map data providers as mentioned above, or from LBG-specific databases such as Niantic Wayfarer.^36^ Another approach is to integrate Spatial Data Infrastructures (SDIs) provided by the public sector. An SDI can be defined as “an enabling platform for data sharing [including] people, data, access networks, institutional policy, technical standards, and human-resources dimensions […], supporting ready access to spatial information” [^37^, p. 727]. The popularity of SDIs and their potential to support various institutions and initiatives has grown over recent years, especially with the rise of concepts such as building information modelling^38^ and digital twins.^33^ Many countries (e.g. Switzerland,^39^ Germany,^40^ and Finland^41^) support a national SDI, however, LBGs require SDIs with global coverage which remain rare. A transnational SDI covering a larger geographic regain is the INSPIRE geoportal,^42^ which is an endeavour of the European Union based on the directive 2007/2/EC of the European Parliament^43^ requiring member states of the EU to provide framework-specific data. Initiatives such as the Global Fundamental Geospatial Data Themes defined by the United Nations Committee of Experts on Global Geospatial Information Management,^44^ advocate for the importance of spatial information and attempt to formalise categories of fundamental geospatial data. Further, the voluntary organisation Open Geospatial Consortium^45^ defines standards and software frameworks that support the interoperability of data sources. Although such initiatives do not directly provide data, they highlight the importance of ever-detailed spatial data as well as a trend towards increasingly accessible and available open data. Finally, we propose a fourth and last trend in the domain of GIS.
– **(GIS) Trend #4:** *Growing data repositories with available and accessible open data curated by institutions and the public sector allow for the generation of increasingly diverse LBG content*

### 2.2 Trends in LBG technical infrastructure and hardware

In addition to GIS and map data, LBGs rely on sensors and computing to augment real-world places with fictional content. More precisely, popular LBGs such as Pokémon GO and Dragonquest Walk^7^ may rely on satellite navigation, pedometers, gyroscopes, mobile hardware and software. An interesting trend here is that new hardware that may be relevant for AR and LBG experiences has been added to the latest phone models. One example comes from Apple, as since iPhone 12 Pro (up until iPhone 15 at minimum), the phones have now been equipped with LiDAR sensors, enabling the creation of point cloud scans of physical objects.^46^ LiDAR also enables the detection of surfaces, which can be useful for locative and AR gaming where this can be harnessed to more precisely and accurately position AR content in the real world.^47^ The technology also has potential in generating holograms of physical objects normally invisible to the user,^48^ a functionality that may be relevant in certain AR game designs. We predict that these advances in AR-related hardware technology help position AR content more precisely in the real world, creating a stronger and more immersive illusion for the user. Thus, we formulate the following trend.

– **(Technical) Trend #1:** *Hardware for AR is moving to a direction that enables more seamless, precise and immersive positioning of AR content within the physical world.*
  In order to afford immersive AR experiences, there is a need to have interfaces and interaction affordances that feel immersive and easy to use. An area of particular focus in this endeavour have been wearable technologies, which utilise a combination of sensors and computing to support AR and gaming experiences.^49^ ^,^ ^50^ Games for smart watches^51^ ^,^ ^52^ can be regarded as one example of the potential of wearables. There are also wearables such as tangible spatially aware objects (e.g., Apple air tags), that are so-far not yet used extensively in the game context, but have the potential to be useful there. With wearables, we have recently seen significant leaps originating from both research (e.g., in the field of medical sciences^53^ ^,^ ^54^) and the industry. For example, in 2023 Apple launched their Vision Pro headset,^55^ which is a type of mixed-reality headset that has a see-through functionality, enabling the display of AR content.^6^ Since head-mounted displays, AR glasses, smart watches, and other wearables are becoming more commonplace, shortcomings related to these devices are being resolved,^56^ and major industry players such as Apple and Meta are investing heavily into them,^6^ we expect the trend of the wearable gaming devices improving only to continue and enable better AR gaming experiences in the future.^50^
– **(Technical) Trend #2:** *Wearable gaming devices will keep getting better, enabling more intuitive interactions and movement in the real world.*
  As outlined in (GIS) Trend #4, we are seeing the enhancement of SDIs, which apart from the aspect of expanding GIS data, also has an infrastructure perspective. Notably, the trend is driven by the concept of Digital Earth and interwoven with the Metaverse notion.^57^ ^,^ ^58^ An SDI includes physical servers, the creation of metadata sets and provision as open data. Masser^59^ identifies the keys of this trajectory as suitable governance structures, facilitating access, building capacity and interoperable data. These deficits might account for why there is currently little access to public sector SDI. However, it is likely that these current deficits will be eliminated, while SDIs will continue to be enabled for increasingly complex data types (see also (GIS) Trend #2). Consequently, we formulate a trend.
– **(Technical) Trend #3:** Spatial data infrastructures are becoming more efficient and will be correspondingly more widely used in LBGs.
  Last but not least, we have the trend of mobile devices getting better and mobile data connections getting faster and cheaper. These trends are rather self-evident, as we see mobile networks being upgraded first from 3G to 4G, and now from 5G to 6G, and we also see newer models for popular brands such as Samsung, Apple and One Plus getting released on a steady pace, each new phone carrying more sensors and computing power than the previous. In addition to devices themselves getting more powerful, we have things such as cloud computing and with a fast mobile connection, more and more of e.g., graphics computing can be offloaded to servers. Such a design has already been introduced e.g., in the Nvidia Shield Tablet,^60^ and these developments are likely to continue as we move to the future. As such, we propose our final two technical trends.
– **(Technical) Trend #4:** *Mobile data connectivity will become better and cheaper.*
– **(Technical) Trend #5:** *Mobile devices will have better capabilities for information processing and computing.*

### 2.3 Trends in design

LBG design has evolved over the years hand in hand with technology. However, there are also certain technology-agnostic design elements that we discuss here.

First, as discussed in the Introduction section, the most popular LBGs such as Pokémon GO or Dragonquest Walk are multiplayer online games.^7^ The social dimension of LBGs has become an integral part of their success, as the games now scaffold social interaction both online and in-person.^15^ ^,^ ^17^ LBGs such as Pokémon GO also strongly feature user-generated content in the form of players submitting and reviewing points of interests (PoIs) for the game,^29^ and since the summer of 2023, now also having the possibility to create walkable paths for others, called Routes. Social playing is emphasised on various levels: there are social in-person gatherings,^17^ online discussions about the game,^61^ coincidental meetups with strangers and motivation to leave one’s house with follow-up potential for meeting and interacting with others.^14^ The social dimensions only appears to have become more important with latest updates and developments in Pokémon GO and other LBGs,^16^ ^,^ ^62^ and thus, we can expect the trend of social playing to continue moving to the future.

**Figure 2:**
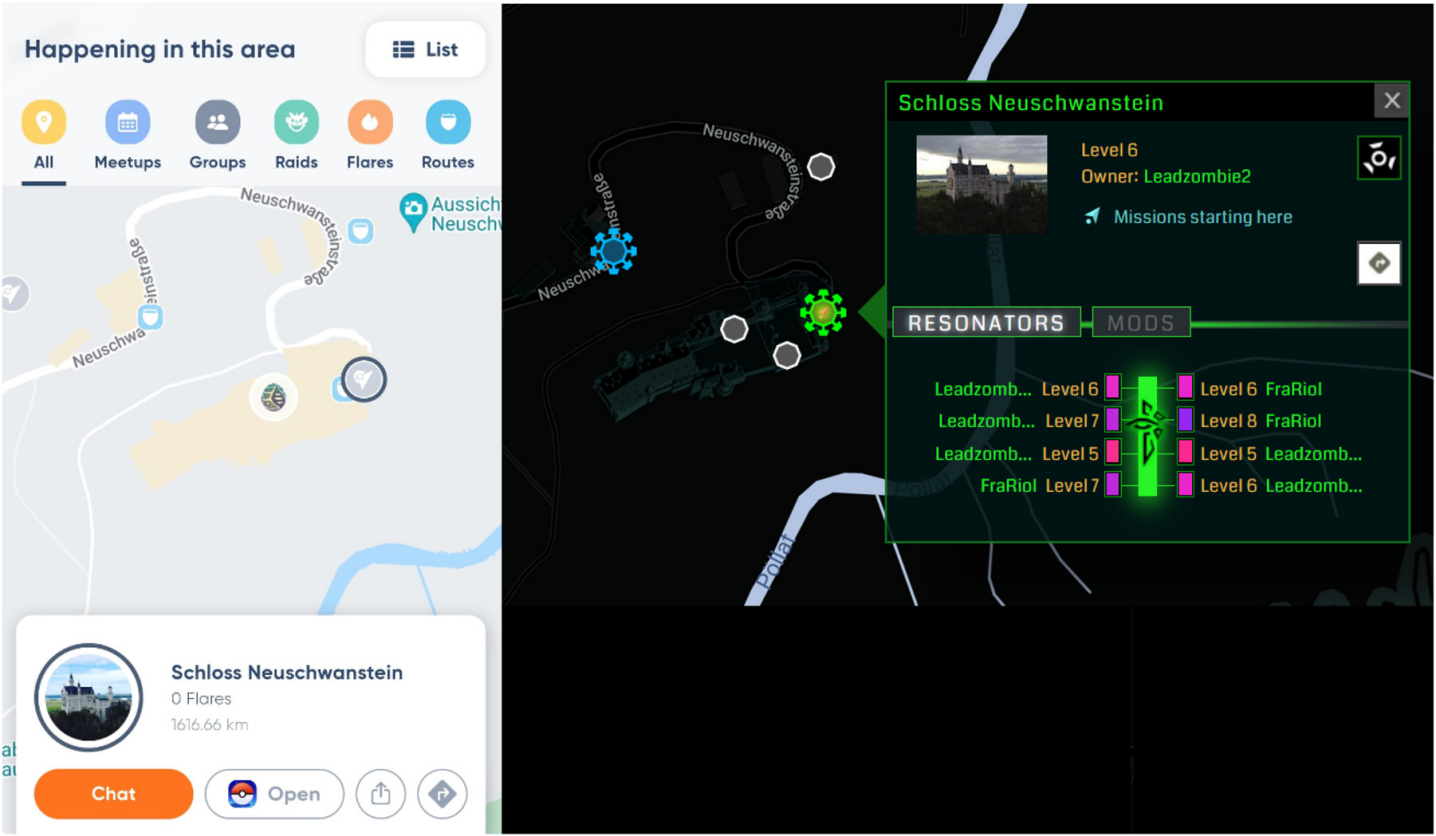
Two screenshots of the Neuschwanstein Castle taken by the authors. On the left we display a screenshot from Niantic Campfire, displaying the Pokémon GO view of the castle. On the right we have a screenshot from the game Ingress, taken by the publicly available Ingress Intel Map service.

– **Design Trend #1:** *In-person social playing is a powerful dynamic that LBG designers will likely know to better harness as the genre moves forward.*
  Second, accelerated by opportunities in map data and hardware, we may see new ways to make use of map data in LBG design. Already today, we notice a few key differences in how LBGs make use of available map data.^1^ ^,^ ^4^ ^,^ ^29^ One such difference is in how contemporary popular LBGs connect in-game points of interests (PoIs) to real-world locations or not.^1^ For example, games such as Jurassic World: Alive and Dragonquest walk contain generic PoIs that are pseudo-randomly distributed across a real-world map, whereas games such as Pokémon GO and Ingress (see Figure 2) have most of the in-game interactable PoIs connected to real-world objects and locations.^1^ One challenge of LBGs is the uneven distribution of POIs, which differs for urban and rural areas, for example.^30^ Algorithms remedying this challenge are being expected.^63^
– **Design Trend #2:** *LBGs design will look to better combine and integrate the games with real-world landscapes, objects and characteristics.*
  Third, we have seen contemporary LBGs add mechanisms that support the games as being part of the players’ daily lives. Mechanisms such as daily first catches in Pokémon GO, daily hacks and hack streaks in Ingress and daily quests in Monster Hunter Now are good examples of popular multiplayer online game mechanics that aim to make playing a routine for the players, a part of their daily schedule. During the COVID-19 pandemic when government measures led LBG designers to alter their game design, we saw a practical example of how the developers aimed to adopt the design of their game in a way that corresponds with players’ lives.^64^ To accommodate the changes in e.g., recommendations to self-isolate, games such as Harry Potter: Wizards Unite and Pokémon GO were changed to include more opportunities for remote and solo play.^64^ When we look at evidence from studies on locative crowdsourcing, we also see that participants contribute mostly after work when commuting,^65^ suggesting that LBGs have become a genre of games specifically serving a purpose of offering “something to do” while carrying out daily locative tasks such as walking the dog, commuting to work or walking to a nearby store for grocery shopping. It makes sense from a financial perspective for LBG developers to draw from this unique position of their games and to increasingly make the games more in-line with players’ daily lives. Further, novel wearables and sensors also enable novel game mechanics which are shaped by the dynamics of movement and location rather than a mobile device such as a smartphone being operated by hand. Game inputs may be generated by movement behaviour, such as entering a building using a specific entrance or crossing the road at certain traffic lights. Smartglasses or projections to the ground may be used for game feedback. In terms of data, dynamic data layers, i.e. data that changes over time, may be integrated into the game, e.g. POIs become those locations where the temperature is most appropriate or the air is cleanest – derived from SmartCity-based sensor networks outside the game.^66^ This concept might also be used to support socially desirable goals, e.g., the temporal or spatial avoidance of substances that are harmful to health, such as nitrogen oxides or fine dust. Thus, we propose our final trend:
– **Design Trend #3:** *Towards designs that integrate with players’ daily lives and societal goals.*

## 3 The future of location-based gaming

We summarize the 12 trajectories that we identified and discussed in the previous Section in Table 2. We offered evidence to support these trajectories, but we cannot directly extrapolate the future of locative gaming based on them. We can, however, engage in follow-up analyses. Therefore, next we discuss the future of LBGs from the following perspectives. First, we reflect the trajectories against previous work on the future of LBGs. Second, we analyse how the trajectories alter or impact the connections between LBGs and the physical world. Third, we discuss how the trajectories impact the interactions in LBGs, predominantly from the perspective of whether and how wearables^49^ ^,^ ^50^ might replace or support the smartphone as the main interaction device. Fourth and finally, we propose theses for the future of LBGs based on our primary analysis, and through speculative design, derive three possible future LBG scenarios.

**Table 2:** Summary of the identified trajectories in location-based gaming and how they are likely to manifest in the future.

Thematic area	Trajectories
GIS	1. Spatial data providers and map data services are increasingly augmented with user-generated content including objective descriptive data as well as subjective individual perceptions of environments and services.
	2. Spatial data providers and map data services are offering spatial information with increasing fidelity resulting in real-world places being depicted on maps with increasing precision, levels of detail and real-world information.
	3. Information about real-world spatially situated entities is being recorded and made available with increasing levels of granularity in machine-readable formats, in particular in terms of the categories an entity belongs to.
	4. Growing data repositories with available and accessible open data curated by institutions and the public sector allow for the generation of increasingly diverse LBG content.
Hardware	5. Hardware for AR is moving to a direction that enables more seamless, precise and immersive positioning of AR content within the physical world.
	6. Wearable gaming devices will keep getting better, enabling more intuitive interactions and movement in the real world.
	7. Spatial data infrastructures are becoming more efficient and will be correspondingly more widely used in LBGs.
	8. Mobile data connectivity will become better and cheaper.
	9. Mobile devices will have better capabilities for information processing and computing.
Design	10. In-person social playing is a powerful dynamic that LBG designers will likely know to better harness as the genre moves forward.
	11. LBGs design will look to better combine and integrate the games with real-world landscapes, objects and characteristics.
	12. Towards designs that integrate with players’ daily lives.

### 3.1 Previously proposed and envisioned paths for the future of location-based gaming

While there are multiple academic studies that contain some perspectives on the future of LBGs as a side outcome (see e.g.,^1^ ^,^ ^14^ ^,^ ^67^), to the best of our knowledge, there is much less work explicitly on the future of LBGs. One of the few studies is the work of Mangnus et al.,^68^ who proposes an interesting LBG experience taking place in Utrecht, Netherlands in the year 2040 called *“Utrecht2040”*. The goal of this app is for players to envision the most sustainable experience of their city in the year 2040, and the authors demonstrated that through playing the LBG, players were prompted to reimagine what their city could be like, particularly from the perspectives of inclusivity and mobility.^68^ This study demonstrates that as LBGs are connected to the real world, they are in a prime position to scaffold meaningful relationships with the physical world around us, and LBGs themselves can act as vehicles for urban design.

In August 2021, John Hanke, the CEO of the major LBG developer Niantic, released an essay where he argued that the metaverse, envisioned as a virtual reality, is a dystopian nightmare.^58^ Hanke argues that abandoning physical life for a virtual one would have grave consequences on society as a whole. His proposed alternative is a location-based augmented reality (AR) metaverse that would aim to support and scaffold people’s everyday lives and experiences as opposed to replacing them. Hanke’s vision^58^ is supported by the work of Mangnus et al.^68^ in the sense that both demonstrate that the inherent connections that LBGs have with the real world help keep players grounded in the physical world and can sustain a desire to care for it. The sustainability perspective is also present in research on smart cities^66^ and playable cities.^69^ In this study, we had less focus on the element of sustainability, but none of the trends or trajectories conflict with it either. Returning to Hanke,^58^ we postulate that simply though scaffolding a deeper connection with our world, LBGs can better sensitize people for real-world problems and issues and empower them to care for our world.

### 3.2 Connections between location-based games and the physical world

A key overarching theme in several identified trends are the increasingly strong connections between real-world entities and their digital representations in location-based games. In other words, the connections between the real world and the fictive game worlds in LBGs are becoming stronger. This can be seen as a function of increasing data availability, increasing data quality and increasing number of users contributing to LBGs. We argue that LBGs offer a unique positive feedback loop in (geospatial) data generation and curation in that LBGs use user contributions to generate game content, in return increasing overall motivation to play LBGs, in the long run leading to increased user numbers and as such more potential contributors. We have seen this happen already in the case of Niantic’s Ingress (launched in 2012), which was used to create the backbone geo data for the much more successful Pokémon GO (launched in 2016) later on.^1^ In light of this, we see LBGs, in combination with advances in mobile device sensor capabilities, becoming valuable crowdsourcing platforms, motivating the collection and curation of real-world entities with ever-increasing quality and fidelity.^70^ Technologies such as LiDAR sensors can help here in creating point clouds from the environments where players play. This trend in crowdsourcing will, in our opinion, further support the broader adoption of the technology in the entertainment and education sectors as well as potentially provide data for other contexts such as policy and decision-making in urban and land use planning.

Relating to the growing strength of connections between the real world and virtual worlds, we see LBGs transitioning from a 2.5 dimensional experience (e.g. virtual 3D objects being placed on a 2D representation of the world in map form) with some AR gimmicks,^47^ to a fully immersive 3D experience including elevation information, digital twins of real-world objects as well as integrating indoor environments and AR content. Seeing the emergence of 3D map tiles, we argue that LBGs will not only adopt but be integral in the creation of the third dimension. This is already hinted at in the popular location-based game developer Niantic adding object scanning features in their games Pokémon GO and Ingress^29^ where users take a series of images of points of interest, which are then used to create 3D models of said objects. We believe true 3D experiences (including indoor gameplay) hold great potential in LBGs and will emerge more broadly as the technology evolves.

Among wearables, as discussed in the (technical) trend #2, tangible spatially aware objects are becoming increasingly popular. Devices such as the Apple Airtag and Samsung SmartTag as well as wearables such as smartwatches and smart glasses situate people and objects at specific locations and have seen widespread adoption. This in return suggests an increasing amount of machine-readable data of spatially situated tangible objects which may be exploited in LBGs. We see that in the future, LBGs are going to capitalise on these technologies by integrating spatially situated devices into the gameplay. For example, spatially aware temperature sensors may situate a player in a respective LBG world as well as manipulate the game world according to the real-world temperature of the respective player. Spatially situated (autonomous) smart devices may become integral to the gameplay (e.g. tangible game characters that are passed on from location to location), further increasing the real-world to virtual-world connections.

### 3.3 Moving away from staring at the smartphone screen

A major consideration in LBGs is the inevitable divided attention between the real-world users are moving through and the virtual game world depicted on their device screen. Users must carefully split their focus between real-world phenomena (e.g. traffic, restricted areas, local laws and regulations, terrain traversability), whilst simultaneously trying to immerse themselves in the fictional world provided by the location-based game. We argue this restricts LBGs from becoming fully immersive experiences. However, through the identified trends, we see LBGs becoming increasingly hybrid in terms of interacting with the game world. In particular, we see two overarching and complementary directions for location-based games in 10 years: (1) the incorporation of alternative interaction possibilities such as LBG specific sensors and wearables; and, (2) a mixed approach to game world exploration through smart devices as well as more computationally powerful devices such as desktops and consoles.

When sticking to the aspect of outdoor locomotion in LBGs, the first trend seems paramount and is supported by wearable devices,^50^ and head-mounted displays such as AR glasses that will shift our focus away from the mobile device to the real world.^6^ When focusing on the second aspect, we notice that LBGs are commonly played on mobile devices with limited computational power compared to other gaming platforms. It is thus possible that a new wave of LBGs will emerge, incorporating a mixed approach to interacting with the game world. We expect to see game worlds based on real-world data, augmented with fictional content. With increasingly immersive capabilities such as VR headsets for exploring virtual landscapes, this form of play may become a significant part of gaming in the near future. There is also a third option, a type of hybrid play where some users may be mobile players in the real world and others explore a digital twin of the real world that dynamically updates according to mobile LBG players’ behaviours.

These new forms of LBG play would greatly increase the exploration of, and interactions with the virtual hybrid digital twin game world, opening up various interesting new avenues in terms of education (e.g. exploring new areas of the world and interacting with educational content outside of one’s area of daily mobility), participatory planning (e.g. allowing users to create 3D suggestions for potential infrastructure as has shown potential with Minecraft^71^) as well as offer an abundance of new gameplay possibilities (e.g. massively multiplayer online role-playing games set in real-world locations or fantastic interpretations of real-world entities). As such, we believe LBGs may address the issue of split attention and limited immersion by moving towards a multi-faceted approach to interacting with the underlying data and by extension, the virtual game worlds.

### 3.4 Proposed theses for the future of LBGs

Based on the trends outlined in Table 2, as well as the above presented follow-up analysis, we propose the following theses that could represent the outcomes of the trajectories (mediated by other factors discussed in our analysis) in 10 years. The theses do not necessarily have to apply to all LBGs, but describe characteristics that may be realised in particular LBGs. The theses are displayed in Table 3.

**Table 3:** Our theses for the future of locative gaming. These theses are based on the trends that we elucidated in this work.

#	In 10 years’ time, there can be LBGs …
1	… that control people’s behaviour without coercion, solely through the fun of playing, in such a way that socially relevant goals can be achieved, for example promoting health far beyond the already present encouragement of movement.
2	… that position fictional content accurately into the physical world, harnessing more elements of the physical world to become part of the playing experience.
3	… that do not require players to focus on an external mobile device screen, but visualise content directly to their field of view via head-mounted displays such as smart-glasses or holo-projections.
4	… that are not solely played through outdoor locomotion, but are to be explored from home via VR headsets, home gaming consoles or PCs. In other words, LBGs that are hybrid worlds building off the digital twin concept where some players might be at home playing with their PCs or VR gear and others moving in the real world with mobile devices.
5	… that integrate dynamic points of interest that are derived from sensor network data, and where actions in the digital world also affect entities in the physical world.
6	… that use open data from the public sector as a key data source.
7	… that are integral to the collection and curation of digital representations of real-world entities.
8	… which support inclusive spatial crowdsourcing as well as bottom-up participatory engagement of citizens in policy and decision-making processes.
9	… that gamify beneficial real-world processes, such as cleaning, taking out the trash or helping a pensioner cross the road.
10	… which rely not only on PoIs, but paths, routes, temperature, elevation, humidity and other measurable and quantifiable real-world elements for creating immersive gameplay.

The first thesis links to advances in technology and design, where we will have fun activities that subtly nudge players to do things that promote their health. We already see this thesis in action in games such as Pokémon GO where players move and get fresh air as a collateral outcome of their playing.^14^ The second thesis links to advances in both GIS and hardware and is the outcome of the trend of LBGs utilising more elements of the real world as part of the games.^1^ The third thesis links to hardware. As we obtain new and better head-mounted displays^6^ and wearables^50^ we get new opportunities for LBG design where the focus shifts away from the smartphone screen and more towards the real world. The fourth thesis is connected to design opportunities arising from advances in GIS and technology, whereby we are able to explore digital twins of real-world cities and landscapes from the comfort of our home in a more immersive and gamified setting. The fifth thesis is the outcome of advances in GIS, hardware and design to some degree, and suggests that instead of relying solely on elements that already exist in the physical realm, LBGs might start harnessing sensor networks and other internet-of-things structures that seem to be becoming more and more popular in urban environments and smart cities. The sixth thesis links to a trend in GIS where open map resources such as OSM are becoming more widespread and of higher quality. We have government bodies and organisations providing a large variety of open data, and it appears to be only a matter of time before LBGs make better use of the available resources. The seventh thesis links to trends in crowdsourcing^29^ ^,^ ^70^ ^,^ ^72^ where LBGs have recently been harnessed to collect real-world data. This is an important potential benefit of playing LBGs, and we see it likely that this element will be leveraged more in upcoming years. Thesis number 8 relates to design and GIS trends, as well as the previous thesis, and suggests that through LBGs and crowdsourcing people can be empowered to participate in shaping their cities, both in the physical and digital spheres. We have already seen examples that hint towards this direction, such as e.g., the work of Morschheuser et al.^72^ who created a prototype game for letting people know about free parking spots. The ninth thesis links back to the beneficial collateral outcomes of playing LBGs, and how through LBGs we might reward and subsequently encourage people to perform tasks and activities that are good for society. Finally, the tenth thesis is related to advances in GIS, and how these may be operationalized in LBG design. We argue that more information of the physical world could be used to create more immersive LBG gameplay.

### 3.5 Three examples of future LBGs

With ten theses now laid out, next we wish to elaborate on how these may be put into practice with concrete examples. Thus, in this section we ideate and present three possible future designs following the speculative design approach.^73^ ^–^ ^75^ These designs illustrate the mechanisms and dynamics in practice and offers a lens to a possible future of the LBG genre. We base two of these designs on the Pokémon franchise^76^ since Pokémon GO is a major reference point in the LBG genre^1^ and the Pokémon franchise is well-known and documented. We argue that for many, Pokémon offers a shared starting point for understanding how the dynamics and ideas presented in this paper could be brought to life. In the third idea we depart from the Pokémon franchise to bring an alternative perspective on these issues through a fictive example game.

#### 3.5.1 Example 1: the entire world is a virtual playground – introducing “Pokémon world”

There have been cases of ambitiously large adventure games, such as The Legend of Zelda, Breath of the Wild which was noted to have a map of the size of the city of Kyoto^77^, The Witcher 3: Wild Hunt with its two expansions, Eve online and No Man’s Sky^78^ that promises an entire universe to explore. With all these games one of the fundamental issues is that to make such large maps populated by playable content, procedural content creation or enormous development teams are required. One of the criticisms that, for example, *No Man’s Sky* received from players according to Steam reviews was, that while the game world was indeed huge, the content and gameplay were repetitive and full of bugs and other usability issues.^78^ As we have argued for in this work, we now have the possibility of transforming the entire world into a virtual playground. This is not to say that this task will be easy and cheap, but it may bring along various advantages (e.g., in relation to experiencing real culture and history). Currently people are already able to move around in Google Maps Street View and Google Earth, and explore our planet at will,^79^ but in the near future, we could explore our world in a more game-like setting, where familiar and famous buildings, structures and landscapes are given new meanings and purposes.

Let’s take the example of Pokémon and imagine it as such a game. The Pokémon anime series and game are built on the notion of “what if this world of ours had creatures called Pokémon in it”.^76^ Now this vision can be brought to life by creating a world-scale virtual Pokémon game that uses a digital twin of our earth to precisely situate virtual content. Players can explore familiar streets, but now with new virtual content added in the mix. The game may not have to include the entire world, but could start with the implementation of the game in one or few popular cities around the world. Players would experience replica of existing streets, buildings and natural locations virtually, but with the addition of fictional Pokémon elements, making the experience more fun and gameful. The game could include cultural and historical information that is accurate to the degree, that playing could be educational. This type of a game would solve issues with procedurally generated content being repetitive and boring, and the use of real-world environments as part of the game world, would enable the learning of real geography, urban culture and history. Players could walk and play as they would in any 3D game and full online multiplayer functionalities could be included. Such an experience would surely offer new frontiers in how we view and understand our world, and we argue that it is only a matter of time before we see the first of these types of games.

#### 3.5.2 Example 2: augmenting the everyday experience – introducing “Pokémon GO 2”

Pokémon GO is maintained as a live service game with incremental updates to the game on a regular basis. Over the years the game has received new features, but the essential mechanics of augmenting a real-world map with fictional content and exploring the world with a focus on collection have remained untouched. Now with improvement in the quality, accuracy and fidelity of GIS we are able to create AR gameplay that more closely links to the physical world, and we will have hardware that supports more seamless interaction not requiring a smartphone through which the AR world is presented to the player. With the new possibilities in mind, we propose a new design for Pokémon GO which we call here “Pokémon GO 2”.

In the next major iteration of the ideas originally introduced to the world in 2016, players will be visualizing the AR world through a head-mounted display as opposed to a smartphone, and interacting with the world through wearable gloves. While a map-based visualization of the world will still be available, the focus here will be on a natural eye-gaze-based view of the world. The Pokémon creatures will appear on the players’ line of sight and the wearable gloves will allow for more natural interactions than a smartphone. Harnessing the latest AR technology and point cloud scans of real-world objects and buildings, AR content could be more accurately positioned within the real world. Harnessing the latest in GIS, we could ensure that poison-type Pokémon appear near polluting factories and sewers, electric-type Pokémon appear near power lines and power plants and ghost-type Pokémon can be found at graveyards. This type of a Pokémon game that more seamlessly connects with the physical world, and which allows more natural interactions with it, would bring the original vision of Satoshi Tajiri closer to life.

The interactions in this game would resemble more closely to what we see in the Pokémon anime,^76^ meaning that throwing a PokéBall would be an actual throw motion, and calling back a Pokémon could similarly be a hand gesture. This would make the game more like an exergame,^80^ as it would contain more full body exercise than the current version of Pokémon GO. There are limits to what this type of a game can present to players, and, for example, animating all Pokémon creatures to realistically move in the real world, and do realistic things (as opposed to just moving back and forth or standing still) will still be an enormous challenge. However, as we move further into the future, it is likely that these types of challenges will be solved.

#### 3.5.3 Example 3: interweaving indoor and outdoor play – introducing “Bird Tales”

In this third scenario we offer an example of a hybrid future scenario between the previous two proposed LBG designs. Traditionally, LBGs are developed to be played outside, making use of a user’s locomotion as a way of navigating the virtual game world.^1^ On the contrary, traditional PC and console video games focus on designed or procedurally generated virtual content where real-world locomotion is not part of the gameplay. In the future, we may see a merging of outdoor location-based gameplay with indoor PC and console gameplay.

An example to demonstrate the interweaving of indoor and outdoor play is a fictive game called “Bird Tales” available both as an LBG on location-enabled smartphones and as a PC game. The PC version of the game can be summarised as an online survival game where players take the role of a specific bird character which they choose from a set of playable bird species. In the open-world sandbox game environment, players are tasked with building a nest, finding food, attacking other players and defending their in-game items from other players. The game world is a digital twin of the real world and to start playing, players must choose a virtual tree in the game as their starting point. The virtual trees in the game are digital twins of real-world trees at their respective coordinates.

Through the location-based gameplay on their smartphones, players have the possibility of scanning nearby single trees or whole forested areas, effectively collecting real-world spatial information (^29^ ^,^ ^70^). The collected scans are, in turn, used to generate the game world, adding new digital twins of real-world trees to the PC version of the game. Playing the LBG thus directly influences the PC version by providing a larger virtual game world to explore, effectively motivating both indoor and outdoor play. In addition, the game hosts a plethora of educational content on various bird species, habitats and ecosystem services whilst generating valuable data for forestry professionals and local policymakers.^29^ This fictive example shows the untapped potential of uniting players drawn towards the outdoor nature of location-based games and players drawn to more sedentary indoor gameplay.

## 4 Conclusions

In this work, we extracted 12 key high-level trends in location-based gaming which we summarise in Table 2. These trends can offer readers an overview of where LBGs as a genre seem to be heading. However, our interpretation presented within this article is but one such perspective, and by no means exhaustive. Based on the extracted trends, we generated 10 theses (see Table 3) of what we believe LBGs are gravitating towards and speculate on the evolution of LBGs in the next ten years. Our view of the future of LBGs is overall positive and aligns in most parts with John Hanke’s arguments that we should rather strengthen the ties between real-world entities and virtual representations in favour of investing in fully virtual abstract worlds as advocated by contemporary metaverse discussions.^58^ In other words: we should make an effort to increase appreciation of being in the real-world instead of retreating fully into the digital. However, we also see the potential of LBGs to cater to both the appreciation of the real and virtual worlds by moving towards multiple forms of interaction possibilities. In particular, we see great potential of LBGs to provide novel forms of underlying spatial data collection through tangible game-relevant devices and nuanced insights into salient features of an individual’s surroundings, in turn offering additional location-based content.

With this research, we contribute to LBG design research and industry practice by offering an overview of current pertinent trends and providing decision-makers with literature based potential future scenarios. Our work can be useful for scholars and decision-makers looking to better understand developments in the fields of playable cities and transurbanism^81^ and human-nature interaction.^14^ ^,^ ^82^ ^,^ ^83^ We offer a trend-based look into the near future, providing a forecast of what locative play could transform in the near future given recent and proposed advances in technology, infrastructure and design innovations.

To conclude this work, we wish to discuss the limitations of this study. First, since we covered a broad topic, there are likely themes and trends that we miss, or did not emphasise strongly enough. For example, there is a stream of research on location-based advertising and how it may play an increasingly bigger role in LBGs.^67^ We did not focus on this aspect and others as we wanted to stick to key trends that, in our view, have the most impact on the future of LBGs. Thus, what we presented, was the authors’ own interpretive reading and understanding of the scholarship on LBGs and what elements within are paramount. Second, as is typical with research looking into the future,^84^ our analysis ultimately is no more than an informed guess based on the identified trends. While this type of work has scientific and practical value through synthesising the current status quo and proposing future directions, the work is imprecise by design, and we encourage scholars to keep studying this topic as we move towards the future of locative gaming. For example, recent advances in lidar technology have enabled new forms of location-based AR interactions that generate data of our surroundings.^85^ Furthermore, we based our analysis on immediate trends that are currently observable, and thus, this work inevitably omits several possible developments that might arise in the field of locative gaming in upcoming years.
